# Smoking as a Cause of Fatty Heart

**Published:** 1864-11

**Authors:** 


### SMOKING AS A CAUSE OF FATTY HEART.

Dr. Henry Kennedy, in a paper read before the Surgical Society of Ireland, on fatty heart, makes the following observations on the influence of tobacco smoking in its production:

"I must notice one (cause of this disease) which has year after year been gradually forcing itself on my attention, till it has now reached the strongest conviction in my mind—I mean the habit of smoking, which, I

believe, I have traced in many instances to have been the predisposing cause of the disease. No one is more aware than myself of the difficulties which beset a question of this sort, nor the great opposition which, for obvious reasons, it is likely to meet. Still, the opinion has not been taken up hastily, nor, as I think, without such proof as the subject admits of. All will recollect that within a very few years a great paper war was carried on in the pages of the *Lancet* on the effects of tobacco, and the opinions expressed were sufficiently contradictory. Amongst them all, however, I did not observe one point noticed which seems to my mind of great importance in this question. It is the fact that if any one, no matter what his temperament may be, gets out of health, so that the powers of his system are lowered, he must then either lessen his smoking or give it up entirely. I have met no exception to this statement, which every one may test for themselves—as, for instance, in cases of paralysis, no matter how slight they may be. From the fact, however, I conclude that tobacco, besides other effects, is a depressor of the nervous system, and that there is a constant antagonism going on between it and the healthy state of the constitution, and when used too freely it ultimately engenders a state of health which is very apt to be followed by a fatty heart. At any rate, whatever the explanation be, the fact is as stated above, and I have seen now too many cases of fatty heart, in what are called heavy smokers, to have any doubt on the matter.

"This 4th March, a case which strongly confirms some of the remarks just made came under my notice, and for the third time. The patient, aged 34, is a man of full height, made in the very finest proportions, and remarkable, or at least was, for great physical strength and activity. He has always been strictly temperate as regards strong drink, but is the heaviest smoker I recollect to have met. About three months since he began, and without any cause he could discover, to lose flesh and strength very rapidly, and his wind, as he called it, became so short that he was compelled to give up active exercise. He now looked pale and depressed, having had a cold, which he found it hard to shake off. He told me he had, at my wish, twice tried active exercise since I last saw him. On the first trial he got through it but badly; on the second he was forced to give it up, as his breathing became so hurried and his heart beat so violently. It seems scarcely necessary to add that he had been driven to give up his darling tobacco.

"Except the pulse, there is nothing in this case to indicate disease The two sounds of the heart are distinct and unattended by murmur. There is no increase of dull sound on percussion, nor can I say that the impulse varies from health. Whilst he sits, however, the pulse beats but 48 in the minute, and it was just the same from the first time I saw him. It is large and full to the finger, under which it passes slowly, and is readily compressed. Any movement at once increases the beats, and more than occurs in the healthy state.

"Now, in this case I have scarcely a doubt that the heart has becomes fatty, and most probably in the worst form; I mean where the muscle itself has degenerated, Yet, he tells me, he passed a physician and had his life insured just five months since!"—[*Dublin Medical Press,* April 20, 1864· *Am. Journal Medical Sciences.*

